# Association between Micronutrient-Related Dietary Pattern and Cognitive Function among Persons 55 Years and Older in China: A Longitudinal Study

**DOI:** 10.3390/nu15030481

**Published:** 2023-01-17

**Authors:** Xiaofan Zhang, Feifei Huang, Jiguo Zhang, Yanli Wei, Jing Bai, Huijun Wang, Xiaofang Jia

**Affiliations:** National Institute for Nutrition and Health, Chinese Center for Disease Control and Prevention, Key Laboratory of Trace Element Nutrition, National Health Commission of the People’s Republic of China, Beijing 100050, China

**Keywords:** cognitive function, dietary pattern, reduced rank regression, China

## Abstract

Appropriate dietary patterns for preserving cognitive function in China remains unknown. This study explored the association between dietary pattern and cognitive function in the Chinese population. A total of 6308 adults aged 55 and above who participated in the China Health and Nutrition Survey at least two waves from 1997 to 2018 were selected. The dietary pattern was determined using the reduced rank regression method with responses regarding vitamin C, vitamin E, zinc, iron, copper, and selenium intakes. We used a three-level random coefficient model to evaluate the association. A “vegetable-pork” dietary pattern characterized by high intakes of Legume products, vegetables, fruits, nuts, pork, fish, and plant oil was identified. Compared to the first quartile (Q1) of dietary scores, the regression coefficients (*p* value) for the global cognitive score across Q2, Q3, and Q4 were 0.27 (*p* = 0.030), 0.45 (*p* < 0.001), and 0.50 (*p* < 0.001), respectively. The adjusted odds ratios (ORs) and the 95% confidence intervals (CIs) for poor cognition across Q2, Q3, and Q4 were 0.82 (0.73 to 0.93), 0.79 (0.69 to 0.91), and 0.74 (0.63 to 0.86), respectively. The relationship appeared to be stronger among people who were 65 years and older, women, people from the south, and smokers. Higher adherence to the “vegetable-pork” diet is associated with better cognitive function among Chinese elders.

## 1. Introduction

Population aging and the burden of dementia are global issues. According to the Global Burden of Disease Study 2016, 43.8 million individuals were living with dementia, which has more than doubled from 1990 to 2016. For people over the age of 70, dementia is the second leading cause of death after ischaemic heart disease [1]. China has 264.02 million people aged 60 years or older, representing 18.74% of the total population of 1.41 billion people [2]. Due to its large number of older adults, China has the highest number of dementia patients in the world, with 15.07 million people aged 60 and above suffering from dementia [3]. However, the current lack of effective treatment for dementia suggests the key role of prevention strategies. It is important to identify modifiable influencing factors to prevent or delay the onset of dementia.

Vitamins and minerals are critical for the structure and function of brain cells, especially during ageing, when significant structural and functional changes in the brain occur [4]. Previous studies have shown that a variety of micronutrients are closely related to cognitive function [5,6,7]. Vitamin C and vitamin E can reduce the deposition and formation of the Aβ protein through their antioxidant effect, which plays an important role in improving cognitive function [6]. Dietary zinc, iron, copper, and selenium intake may be negatively correlated with the prevalence of a lower cognitive ability [7]. Different from a single nutrient or food, dietary patterns reflect the synergistic or antagonistic effects of different foods and nutrients and have a greater impact on health.

Many studies have been conducted on dietary patterns and cognitive function, while most of them are based on prior methods and specific dietary patterns applicable to western populations, such as the Mediterranean diet (MeDi) [8] and the Dietary Approaches to Stop Hypertension diet (DASH) [9], which were associated with the reduction of mild cognitive impairment (MCI) and dementia. These diets are very different from the traditional Asian diet, so it is important to find beneficial dietary patterns specific to Asian populations. There is limited evidence on the relationship between micronutrient-related dietary patterns and cognitive function based on reduced rank regression (RRR), and there are even fewer longitudinal studies. A cross-sectional study from the United States reported that an iron-related dietary pattern was associated with lower cognitive function among adults aged 60 years or older [7]. Another study from Korea revealed that a vitamin B6-, vitamin C-, and iron-related “seafood and vegetable” dietary pattern could reduce the risk of MCI among older Korean adults aged 65 years and older [10]. We aimed to explore the longitudinal association between micronutrient-related dietary patterns and cognitive function in the Chinese population aged 55 and above, using data from the China Health and Nutrition Survey (CHNS) between 1997 and 2018, to provide a scientific basis for the development of prevention strategies for dementia.

## 2. Materials and Methods

### 2.1. Study Participants

Data were collected from six rounds of the CHNS in 1997, 2000, 2004, 2006, 2015, and 2018 (the first assessment on participants’ cognitive function was performed in 1997). CHNS is a prospective and ongoing cohort study established in 1989, of which the latest round was conducted in 2018. It used a multistage random-cluster process to recruit the samples in 15 provinces from northeast to southwest of China. Details on study design and procedures were described previously [11]. Cognitive assessments were conducted among CHNS participants who were community dwellers aged 55 years or older. In this study, we excluded individuals with fewer than 2 waves of cognitive measurements, abnormal dietary energy intake (males: energy intake 6000 kcal/d or 800 kcal/d; females: energy intake 4000 kcal/d or 600 kcal/d), abnormal BMI (15.0 kg/m^2^ or 40.0 kg/m^2^), and abnormal or missing dietary information between 1997 and 2018. Finally, 6308 participants aged 55 and above with 16,065 observations were included in our analysis. The number of visits (n) ranged from 2 to 6 per participant: 2 visits (n = 4221), 3 visits (n = 1085), 4 visits (n = 699), 5 visits (n = 246), and 6 visits (n = 57).

The CHNS was approved by the Institutional Review Board of the University of North Carolina at Chapel Hill, Chapel Hill, North Carolina, United States (No. 07-1963), and the Institutional Review Committee of the National Institute for Nutrition and Health, Chinese Center for Disease Control and Prevention, Beijing, China (No. 201524). All participants provided written informed consent prior to the surveys.

### 2.2. Cognitive Function Measurements

The cognitive screening items used in CHNS included a subset of items from the Telephone Interview for Cognitive Status–modified [12]. The screening test was conducted face-to-face during home visit. The screening included immediate and delayed recall of a 10-word list (score 10 for each), counting backward from 20 (score 2), and subtracting 7 consecutively (score 5). A total verbal memory score was calculated as the sum of the immediate and delayed 10-word recall, ranging from 0 to 20. The global cognitive score was the sum of all item scores, ranging from 0 to 27. A higher global cognitive score represents a better cognition. We also constructed an alternative composite score by averaging z scores of verbal memory items and the other items assessing attention and calculation. This alternative scoring helps to reduce the influence of the memory component, which has additional points and is more influential in the global score than other domains [13]. 

The cognitive function test started with the immediate recall of a 10-word list. The investigator (i.e., trained health worker) read ten words at a speed of 2 s per word. After all the ten words were said, the participants were given 2 min to recall these words. For each correctly recalled word, a score of 1 was given. The participants were then asked to count back from 20 to 1. If the participants made a mistake in the first try, a second chance was given. A score of 2 was given to those who answered correctly in the first try, or 1 in the second try. After the count test, the participants were asked to do five consecutive subtractions of 7 from 100. Each correct subtraction was given a score of 1. Finally, the participants were asked to recall the 10-word list tested before. Each recalled word was given a score of 1. In our study, we choose the first quintile of the global cognitive score as poor cognitive function [14], which corresponds to a global cognitive score cut off < 9.

### 2.3. Dietary Measurements

All food intakes, except for water and tea for individual household member aged 2 years and more, including meals and snacks, were obtained through a 24 h dietary recalls for 3 consecutive days (one weekend day and two weekdays). Trained and qualified investigators used food pictures, food models, and a food diary to assist respondents in recalling their dietary intakes in past three 24 h so as to reduce recall bias [15]. The consumption of food condiments (e.g., edible oils and salt) during food preparation and cooking at the household level was weighed during the same 3-day period. The investigator completed and checked the daily dietary survey data, and if there was any doubt, he/she would return to the survey household for further inquiry and confirmation to ensure the survey quality. Detailed dietary data collection and allocation have been described elsewhere [16,17].

According to the Chinese food composition table, we calculated the intakes of various foods, nutrients, and total dietary energy per day. In this study, all foods were classified into 26 items, such as rice, wheat, other cereals, etc., according to the food composition list. Among them, cakes, drinks, fast foods, wine, and starch were excluded due to partial omissions from 1997 to 2018; dried beans and offal were also excluded, with a consumption rate of less than 10%. The final dietary patterns were extracted using 19 food groups, as shown in Table 1.

### 2.4. Covariates

In our analysis, we included several confounders related to diet and cognition, including demographics, socioeconomics, lifestyles, and health indicators collected by questionnaire survey and physical measurement. Demographic and sociological indicators included age, sex, education, employment status, geographic region, urbanization index, and annual household income per capita. The region was divided into south and north. Urbanization index [18] and per capita annual household income were divided into three groups by tertiles. Lifestyles included smoking, alcohol consumption, physical activity, and total dietary energy intake. Smoking an average of at least one cigarette per day before or during survey period was defined as a smoker, otherwise the respondent was classified as a non-smoker. Smokers were further classified as former or current smokers based on whether the respondents still smoked. If the respondents had not drunk alcohol in the past year, we considered them as non-drinkers, otherwise the respondent was classified as a drinker. For drinkers, they were further divided into <3 times/week or ≥3 times/week, based on their drinking frequency. The weekly metabolic equivalent of physical activities was calculated according to the physical activity questionnaire, including traffic, occupational, leisure, and housework physical activities, and grouped into three groups by tertiles in the analysis. Health indicators included obesity, hypertension, diabetes, myocardial infarction, and stroke. Height and weight were obtained by physical measurement, we calculated BMI (kg/m^2^) as weight in kg divided by height in meters (m) squared, and defined obesity if BMI ≥ 24.0 kg/m^2^ according to the Chinese adult’s weight criteria (WS/T 428-2013). According to the definition of hypertension in Guidelines for Prevention and Treatment of Hypertension in China (2018 revised edition) [19], combined with field measurement and disease history inquiry, averaged systolic blood pressure of three measurements ≥ 140 mmHg and/or diastolic blood pressure ≥ 90 mmHg, or those who took antihypertensive drugs within two weeks, or were diagnosed as hypertension by doctors, were determined as hypertension. Diabetes, myocardial infarction, and stroke were assessed based on disease history investigation.

### 2.5. Statistical Analysis

Micronutrient-related dietary patterns were constructed using reduced rank regression (RRR) with the intakes of 19-collapsed food groups as input variables. PROC PLS statement in SAS 9.4 was used to conduct RRR analysis using vitamin C, vitamin E, zinc, iron, copper, and selenium intakes as the response variables. The dependent variables (six nutrients) were non-normal distribution after normality test, so Box-Cox transformation was performed on them. As there were six response variables, six micronutrient-related dietary patterns were extracted. Dietary pattern 1 with the highest explanation was selected as the target one for subsequent analysis. For longitudinal data, we first selected dietary data in 2018 to conduct RRR and extracted the food groups (absolute value of factor load ≥0.25) included in the dietary pattern 1. Then, the factor loads of the selected foods in 2018 were used as weights, and the intake of these foods in all waves were normalized (original intake value-mean/standard deviation). Finally, the sum of the products of the standardized intakes of these foods and their corresponding weights was used as dietary pattern score for each wave. Dietary pattern score in each wave was divided into four groups by quartiles.

The differences in baseline characteristics of the samples by quartiles of dietary pattern score were described and analyzed. The continuous variables were described as mean and standard deviation. Because they did not conform to normal distribution, the rank-sum test was used to analyze the differences between groups. Categorical variables were described by the number of cases and percentage, among which dichotomous variables were analyzed by chi-square test and trend analysis, and ordinal multiple categorical variables were analyzed by rank-sum test. 

For longitudinal data, we used a three-level random coefficient model to evaluate the relationship between dietary pattern score and cognitive function, where time was level one, individual was level two, community was level three, and age was the random coefficient. The traditional statistical methods would ignore the characteristics of the data hierarchy, but because the multi-level model could deal with the unbalanced and incomplete data well, did not require the independence of the data, and took into account the variation between the data levels, the analysis results were more reliable. We used variance component model with random coefficients and logistic model with random coefficients to analyze the association of dietary pattern scores with continuous variables (global cognitive score and its z score and total verbal memory score and its z score) and the categorical variable (poor cognitive function or not), respectively, and adjusted covariates in the models. The steps of model fitting were as follows: First, a three-level empty model with only intercept term was established, and according to the results of covariance test, it was judged whether these data were suitable for multi-level model and whether the setting of each level was appropriate. Then, the random intercept model was fitted by adding explanatory variables. Finally, age may have a random effect on the model, and so it was taken as a random slope to further fit the random coefficient model. According to the test results of the random coefficient, it was judged whether it was suitable. We performed all statistical analyses using SAS 9.4 (SAS Institute Inc., Cary, NC, USA). We considered a two-sided *p* value < 0.05 as statistically significance.

## 3. Results

### 3.1. Micronutrient-Related Dietary Pattern

Six micronutrient-related dietary patterns were driven by RRR methods. Among them, pattern 1 with vitamin C, vitamin E, zinc, iron, copper, and selenium as response variables was characterized by high loadings of legume products, vegetables, fruits, nuts, pork, fish, and plant oil (Table 1). The factor loadings of vegetables and pork rank first and second, so we called dietary pattern 1 the “vegetable-pork” pattern. This pattern explained 37.39% of the response variables, the most highly explained dietary pattern among the six dietary patterns. Thus, the “vegetable-pork” pattern was included in the subsequent analysis as the target diet.

### 3.2. Baseline Characteristics by Quartiles of Dietary Pattern Score

Table 2 showed the characteristics among participants attending the first cognitive function test by quartiles of the “vegetable-pork” dietary score. Across the quartiles of the score, the mean global cognitive score, verbal memory score, and the intake of energy increased. “Vegetable-pork” dietary pattern was positively associated with males, the south, education level, smoking, alcohol intake, urbanization index, family income, overweight–obesity, and was inversely associated with age.

### 3.3. Associations between Dietary Pattern and Cognitive Function

“Vegetable-pork” pattern was related to an improved global cognitive function in a dose–response manner (Table 3). In the fully adjusted model, across quartiles of the dietary pattern score, the regression coefficients (*p* value) were: 0.27 (*p* = 0.030), 0.45 (*p* < 0.001), and 0.50 (*p* < 0.001), respectively. This similar positive association between the “vegetable-pork” pattern and improved z scores of global cognitive functions was observed. No association between dietary pattern and verbal memory score was found in the variance component model with random coefficients after adjusting for all covariates.

### 3.4. Associations between Dietary Pattern and Poor Cognitive Function

Adjusted odds ratios (ORs) and the 95% CIs for poor cognitive function across the quartiles of the “vegetable-pork” pattern score are presented in Table 4. Overall, with the increase in the dietary pattern score, the risk of poor cognitive function in the older population decreased. Older adults in the second, third, and fourth quartiles of the “vegetable-pork” pattern score were less likely to have poor cognitive function compared to those in the lowest quartile. The adjusted OR and 95%CI values were 0.82 (0.73 to 0.93), 0.79 (0.69 to 0.91), and 0.74 (0.63 to 0.86), respectively, after controlling for covariates. We then further stratified the analysis by age, gender, region, and smoking status, and found different results. The relationship between dietary pattern scores and cognitive function appeared to be stronger among people who were 65 and older, women, people from the south, and smokers.

## 4. Discussion

In this study, six cognition-related micronutrients (vitamin C, vitamin E, zinc, iron, copper, and selenium) were used as the dependent variables to obtain a “vegetable-pork” dietary pattern with the highest explanatory degree in the Chinese population aged 55 years and older, characterized by a higher intake of legume products, vegetables, fruits, nuts, pork, fish, and plant oil. Longitudinal data from the CHNS over twenty-one years were used to obtain the longitudinal relationship between micronutrient-related “vegetable-pork” dietary patterns and cognitive function. We found that the higher the “vegetable-pork” dietary pattern score, the higher the global cognitive function score and the lower risk of poor cognitive function. Additionally, the observation of a lower risk of poor cognition was more pronounced among people aged 65 and older, women, southerners, and smokers. These findings could provide a scientific basis for the prevention of cognitive impairment in the older population in China.

To date, many original studies and meta-analyses have reported individual associations between different dietary nutrients and cognitive function [5,6,7]. However, only few studies focused on the combined effects of several nutrients on cognition [10,20]. For instance, a cross-sectional study using 2311 older Chinese individuals [20] showed that saturated fatty acid- (SFA), monounsaturated fatty acid- (MUFA), polyunsaturated fatty acid- (PUFA), and vitamin C-derived food patterns were significantly correlated with higher cognitive function. The total diet approach is an important tool in nutrition research to capture the inherently complex nature of food intake. Dietary patterns can capture any synergistic effects between different foods and nutrients, and the cumulative exposure to foods and nutrients may also lead to stronger associations with health outcomes [21]. There are two main approaches used to derive dietary patterns: a priori or pre-defined dietary patterns, and a posteriori or data-driven/exploratory dietary patterns [22]. Reduced rank regression (RRR) is one of the most widely used exploratory approaches. It uses a prior knowledge about nutrients or biomarkers that have established links to disease to help identify data-driven food combinations in a specific population [23]. Hence, RRR is particularly appealing when the aim is to identify dietary patterns in association with disease endpoints. In this study, we used RRR to obtain a dietary pattern representing six cognitively related micronutrients, which could not only explore the combined effects of different foods and nutrients on cognition, but also reflects the existing eating habits of the study population. 

The “vegetable-pork” dietary pattern obtained in this study may be a healthy dietary pattern suitable for the older population in China, which could reduce the decline in cognitive function. At present, the main dietary patterns associated with Alzheimer’s disease (AD)-related cognitive impairment are the MeDi, DASH, and MIND (the Mediterranean-DASH Diet Intervention for Neurodegenerative Delay) diets. Substantial epidemiological studies [8,24,25,26,27] supported that higher adherence to the above three dietary patterns are associated with a slower decline in cognitive abilities and a greater reduction of incidences of AD. The “vegetable-pork” dietary pattern in this study shared some of the same food components as these internationally recognized dietary patterns, including fruits, vegetables, legumes, nuts, and fish, which are all cognitively beneficial foods. Studies on the relationship between foods, food-groups, and late-life cognitive disorders also confirmed these foods cognitive benefit [28,29,30,31,32]. For example, the consumption of at least one serving of fish per week was associated with a slower cognitive decline among Chinese adults aged 65 and older [28]. Fruit and vegetable intake ≥ 400 g/day was associated with a reduced risk of cognitive impairment among the Brazilian elderly population aged 65 or over [30]. A higher intake of legumes and nuts was related to better performance in global cognition, language, and visuospatial cognitive domains among individuals aged 70–90 from Sydney [32]. Given the lower consumption of olive oil, red wine, low-fat milk, and whole grains among the Chinese population, these foods were not included in the Chinese healthy dietary pattern identified in present study. Surprisingly, the dietary pattern in this study included pork, which was generally not recommended in other healthy dietary patterns. Red meat, a classic element of the Western diet, has been linked to poorer cognitive performance in several studies [33,34]. However, data from recent human intervention trials conducted worldwide demonstrated that daily supplementation of carnosine and anserine (both are imidazole dipeptides, which are abundant in pork) can improve memory loss and reduce the risk of developing AD in older adults [35,36,37,38]. Moreover, pork is the most commonly consumed meat in China, and so a healthy dietary pattern that includes pork is more likely to be followed sustainably in the long term. Findings from a recent, random-controlled trial indicated that, a Mediterranean diet that includes fresh lean pork can be adhered to by non-Mediterranean older adults with positive cognitive outcomes [39]. Therefore, more population studies are needed to explore the role of pork in dietary patterns in cognition.

In this study, we found that the higher women’s adherence to the “vegetable-pork” pattern, the better their cognitive function; however, this association was not found in men. This interaction between diet and gender on cognitive function has been shown in previous studies [40,41,42]. A cross-sectional population-based study in France [41] also investigated sex differences in the diet-cognition relationship in later adulthood. In the healthy dietary pattern that was negatively associated with MCI, food components varied slightly by gender, including the intake of fruits and vegetables in women and the intake of fish and seafood in men. Findings from the NuAge study [42] showed that a sex-dependent relationship may exist between dietary pattern adherence and cognitive function in later life, and compared to women, men may be more sensitive to the effects of an unhealthy Western dietary pattern. Further explanations may come from gender-based differences in biophysiological responses to dietary intake [43,44]. The present study also found that the “vegetable-pork” pattern had a greater protective effect on cognitive function among people 65 and older, southerners, and smokers, which suggested that age, region, and smoking may influence the role of dietary patterns in cognition. Thus, stratification should be considered for the future research.

There are several advantages to this study. First of all, this study combined six micronutrients using the RRR method to drive a new and healthy dietary pattern suitable for the prevention of cognitive impairment in the older population in China, which has not been found in previous studies. Second, since dietary patterns and cognitive function may change over time, this study can better capture the longitudinal changes of dietary patterns and cognitive function by using longitudinal data of CHNS of up to 21 years. In addition, the stratified analysis provided some new findings, suggesting that age, sex, region, and smoking status may influence the association between dietary patterns and cognition. Nonetheless, this study also has some limitations. First, self-reported dietary information may be subject to memory and recall issues. However, this is also common in other relevant studies. In this study, dietary data were collected face-to-face in households and combined with food pictures, meal records, and other tools to minimize bias. Second, dietary pattern extraction can also include more dietary nutrients, such as vitamin D, B vitamins, folic acid, etc., which are also related to cognitive function. The present study did not perform as well due to the lack of related data of these micronutrients in the Chinese food composition table.

## 5. Conclusions

The “vegetable-pork” pattern was significantly associated with reduced poor cognitive function in middle-aged and older Chinese adults. It may be a healthy dietary pattern suitable for the Chinese people to prevent cognitive decline and dementia. We also found that the relationship between dietary pattern and cognitive function appeared to be stronger in people over 65, women, southerners, and smokers. Future prospective cohort studies are warranted to examine the effects of “vegetable-pork” dietary pattern on reducing the risk for the development of MCI and dementia.

## Figures and Tables

**Table 1:** Factor loadings of vitamin C, vitamin E, zinc, iron, copper, and selenium-related dietary patterns based on reduced rank regression (RRR) in Chinese adults aged ≥55 years old.

Food Groups ^1^	Dietary Pattern 1 ^2^	Dietary Pattern 2	Dietary Pattern 3	Dietary Pattern 4	Dietary Pattern 5	Dietary Pattern 6
Rice	0.16	−0.13	0.18	−0.67	0.26	0.09
Wheat	0.21	−0.08	−0.22	0.34	−0.26	0.53
Other cereal	0.15	0.05	−0.07	−0.12	−0.40	0.05
Tuber	0.16	0.22	0.37	0.14	−0.08	−0.01
Legume products	**0.28**	0.16	0.00	−0.21	−0.37	−0.03
Vegetables	**0.45**	0.15	0.57	0.08	0.26	0.15
Fruits	**0.29**	0.16	0.27	0.19	−0.18	−0.34
Salted vegetables	0.07	0.01	−0.02	−0.14	−0.10	0.16
Nuts	**0.25**	0.08	−0.01	−0.01	−0.28	−0.33
Pork	**0.32**	−0.34	0.05	−0.21	0.44	−0.21
Other livestock meat	0.20	−0.23	−0.05	−0.21	0.02	−0.37
Poultry	0.15	−0.13	−0.06	0.02	0.08	0.03
Milk	0.10	−0.05	−0.01	0.03	−0.03	−0.08
Eggs	0.23	−0.09	−0.07	0.24	−0.09	0.14
Fish	**0.29**	−0.35	−0.23	0.38	0.07	−0.27
Plant oil	**0.31**	0.68	−0.50	−0.01	0.31	0.03
Animal oil	−0.01	−0.04	0.15	−0.09	0.19	0.27
Sugar	0.04	0.03	−0.12	0.00	−0.10	−0.10
Salt	0.22	0.27	−0.15	−0.07	0.10	0.26
Explained variation in:						
Food groups (%)	**7.65**	5.38	5.08	5.61	7.06	5.56
Responses (%)	**37.39**	8.38	5.62	2.39	0.74	0.33

^1^ When extracting dietary patterns, excluded foods with a consumption rate of less than 10% include dried beans and offal. Cakes, drinks, fast foods, wine, and starch were also excluded due to partial omissions in the data from 1997 to 2018. ^2^ Dietary pattern 1 with the highest interpretation was selected as the simplified dietary pattern for subsequent analysis.

**Table 2:** Baseline characteristics of Chinese adults aged ≥55 years old by quartiles of “vegetable-pork” dietary pattern score (N = 6308).

Parameters	Q1(n = 1466)		Q2(n = 1651)		Q3(n = 1568)		Q4(n = 1623)		*p*-Value
	n	%	n	%	n	%	n	%	
Survey year									
1997	242	16.5	225	13.6	212	13.5	238	14.7	0.19
2000	151	10.3	220	13.3	172	11	159	9.8	
2004	233	15.9	275	16.7	287	18.3	256	15.8	
2006	99	6.7	119	7.2	137	8.7	105	6.5	
2015	741	50.6	812	49.2	760	48.5	865	53.3	
Age (years)									
55–64	944	64.4	1155	69.9	1152	73.5	1209	74.5	<0.001
65–74	408	27.8	399	24.2	328	20.9	353	21.7	
≥75	114	7.8	97	5.9	88	5.6	61	3.8	
Gender									
Male	587	40.0	769	46.6	776	49.5	888	54.7	<0.001
Female	879	60.0	882	53.4	792	50.5	735	45.3	
Region									
North	783	53.4	642	38.9	481	30.7	434	26.7	<0.001
South	683	46.6	1009	61.1	1087	69.3	1189	73.3	
Education									
Elementary and below	983	67.1	1008	61.1	893	56.9	774	47.7	<0.001
Junior high	277	18.9	331	20.0	332	21.2	398	24.5	
High school and above	206	14.0	312	18.9	343	21.9	451	27.8	
Smoking									
Non-smokers	1068	73.3	1163	70.7	1076	68.9	1084	66.9	<0.001
Former smokers	38	2.6	69	4.2	50	3.2	70	4.3	
Current smokers	351	24.1	412	25.1	435	27.9	467	28.8	
Drinking									
None	1116	76.7	1184	72.1	1075	68.9	1060	65.6	<0.001
<3 times/week	178	12.2	217	13.2	215	13.8	221	13.7	
≥3 times/week	161	11.1	241	14.7	271	17.3	335	20.7	
Employment									
No	960	65.7	1069	64.8	1007	64.2	1018	62.8	0.400
Yes	502	34.3	581	35.2	561	35.8	603	37.2	
Urbanization index									
Low	538	37.2	485	29.7	353	22.8	281	17.5	<0.001
Medium	448	31.0	494	30.3	509	32.8	460	28.7	
High	459	31.8	652	40.0	689	44.4	862	53.8	
Family income									
Low	533	37.0	519	32.0	381	24.5	289	17.9	<0.001
Medium	409	28.4	498	30.7	534	34.3	529	32.9	
High	497	34.5	605	37.3	642	41.2	792	49.2	
Physical activity									
Low	653	44.6	725	43.9	665	42.4	716	44.2	0.199
Medium	374	25.5	470	28.5	479	30.6	532	32.8	
High	438	29.9	456	27.6	423	27.0	373	23.0	
Overweight–obesity									
No	859	58.6	946	57.3	848	54.1	794	48.9	<0.001
Yes	607	41.4	705	42.7	720	45.9	829	51.1	
Hypertension									
No	804	54.8	899	54.4	902	57.5	868	53.5	0.124
Yes	662	45.2	752	45.6	666	42.5	755	46.5	
Diabetes									
No	1372	94.0	1535	94.0	1468	94.5	1505	93.4	0.590
Yes	87	6.0	98	6.0	85	5.5	107	6.6	
Myocardial infarction									
No	1445	99.0	1626	99.3	1544	99.0	1604	99.1	0.832
Yes	14	1.0	12	0.7	16	1.0	14	0.9	
Stroke									
No	1431	98.6	1607	98.2	1532	98.4	1582	98.1	0.734
Yes	21	1.4	30	1.8	25	1.6	31	1.9	
	Mean	SD	Mean	SD	Mean	SD	Mean	SD	*p*-value
Global cognitive score	14.3	6.0	15.1	5.8	15.7	5.9	16.4	5.7	<0.001
Verbal memory score	9.5	4.8	9.9	4.7	10.2	4.8	10.7	4.8	<0.001
Dietary energy(kcal/d)	1760.6	618.9	1940.8	559.8	2144.4	629.8	2592.7	752.6	<0.001

**Table 3:** Regression coefficients (*p* value) for cognitive function by quartiles of “vegetable-pork” pattern score among Chinese adults aged ≥55 years old attending China Health and Nutrition Survey between 1997 and 2018.

	Q1(ref)	Q2	Q3	Q4
Global cognitive score				
Model 1 ^1^	0.00	0.64(<0.001)	1.16(<0.001)	1.73(<0.001)
Model 2 ^2^	0.00	0.39(0.001)	0.67(<0.001)	0.92(<0.001)
Model 3 ^3^	0.00	0.27(0.030)	0.45(<0.001)	0.50(<0.001)
Verbal memory score				
Model 1 ^1^	0.00	0.38(<0.001)	0.66(<0.001)	1.06(<0.001)
Model 2 ^2^	0.00	0.21(0.046)	0.33(0.002)	0.50(<0.001)
Model 3 ^3^	0.00	0.11(0.291)	0.13(0.228)	0.12(0.336)
Global cognitive function Z score				
Model 1 ^1^	0.00	0.09(<0.001)	0.16(<0.001)	0.23(<0.001)
Model 2 ^2^	0.00	0.06(<0.001)	0.10(<0.001)	0.13(<0.001)
Model 3 ^3^	0.00	0.05(0.002)	0.08(<0.001)	0.09(<0.001)
Verbal memory Z score				
Model 1 ^1^	0.00	0.15(<0.001)	0.25(<0.001)	0.41(<0.001)
Model 2 ^2^	0.00	0.08(0.046)	0.13(0.002)	0.19(<0.001)
Model 3 ^3^	0.00	0.04(0.288)	0.05(0.230)	0.05(0.340)

^1^ Regression coefficients and *p* values were estimated with random-coefficient regression models (age as the random coefficient) with three levels (time, individual, and community). ^2^ Model 1 did not adjust any variable and only included quartiles of dietary pattern score. ^3^ Model 2 adjusted for survey year, age, gender, region, education, employment, urbanization index, and family income. Model 3 further adjusted for smoking, drinking, physical activity, overweight–obesity, hypertension, diabetes, myocardial infarction, and stroke.

**Table 4:** Adjusted odds ratios (95% CIs) for global cognitive score below nine across quartiles of “vegetable-pork” pattern score among Chinese adults aged ≥55 years old attending China Health and Nutrition Survey between 1997 and 2018.

	Q1(ref)	Q2	Q3	Q4
Overall ^1^	1.00	0.82(0.73 to 0.93)	0.79(0.69 to 0.91)	0.74(0.63 to 0.86)
Age (years) ^1^				
55–64	1.00	0.84(0.68 to 1.05)	0.80(0.64 to 1.01)	0.83(0.64 to 1.08)
65–74	1.00	0.89(0.74 to 1.07)	0.80(0.65 to 0.98)	0.68(0.53 to 0.86)
≥75	1.00	0.66(0.51 to 0.86)	0.72(0.54 to 0.96)	0.71(0.49 to 1.01)
Gender ^2^				
Men	1.00	0.84(0.68 to 1.04)	0.86(0.69 to 1.07)	0.83(0.65 to 1.07)
Women	1.00	0.82(0.71 to 0.96)	0.76(0.64 to 0.91)	0.69(0.56 to 0.85)
Region ^2^				
North	1.00	0.94(0.77 to 1.15)	0.87(0.69 to 1.10)	0.72(0.54 to 0.96)
South	1.00	0.75(0.64 to 0.88)	0.75(0.64 to 0.89)	0.74(0.61 to 0.90)
Smoking ^2^				
No	1.00	0.90(0.78 to 1.04)	0.80(0.68 to 0.94)	0.78(0.64 to 0.93)
Yes	1.00	0.64(0.49 to 0.83)	0.79(0.61 to 1.03)	0.66(0.49 to 0.89)

^1^ Odds ratios and 95% CIs were estimated with random-coefficient regression models (age as the random coefficient) with three levels (time, individual, and community). ^2^ Adjusted for age, gender, region, education, smoking, drinking, employment, urbanization index, family income, physical activity, overweight–obesity, hypertension, diabetes, myocardial infarction, stroke, and dietary energy. Removed corresponding stratified variable from the adjusted covariates mentioned above.

## Data Availability

Data sharing is not applicable to this article.
